# Nutritional Markers and Body Composition in Hemodialysis Patients

**DOI:** 10.1155/2015/695263

**Published:** 2015-01-11

**Authors:** Rodolfo Valtuille, Maria Elisa Casos, Elmer Andres Fernandez, Adrian Guinsburg, Cristina Marelli

**Affiliations:** ^1^FME Burzaco, 2289 Espora Avenue, Burzaco, B1852FZD Buenos Aires, Argentina; ^2^Universidad Católica de Cordoba, 3555 Armada Argentina Avenue, X5016DHK Córdoba, Argentina; ^3^FMC Argentina, 707 Arenales Street, C1061AAA Buenos Aires, Argentina

## Abstract

The aims of this study were to analyse body composition, to detect the presence of undernutrition, and to establish a relationship between undernutrition and the biological markers routinely used as indicators of nutritional status in hemodialysis (HD) patients (pts). We used a body composition monitor (BCM) that expresses body weight in terms of lean tissue mass (LTM) and fat tissue mass (FTM) independent of hydration status. From nine HD units, 934 pts were included. Undernutrition was defined as having a lean tissue index (LTI = LTM/height^2^) below the 10th percentile of a reference population. Biochemical markers and parameters delivered by BCM were used to compare low LTI and normal LTI groups. Undernutrition prevalence was 58.8% of the population studied. Low LTI pts were older, were significantly more frequently overhydrated, and had been on HD for a longer period of time than the normal LTI group. FTI (FTI = FTM/ height^2^) was significantly higher in low LTI pts and increased according to BMI. LTI was not influenced by different BMI levels. Albumin and C-reactive protein correlated inversely (*r* = −0.28). However neither of them was statistically different when considering undernourished and normal LTI pts. Our BCM study was able to show a high prevalence of undernutrition, as expressed by low LTI. In our study, BMI and other common markers, such as albumin, failed to predict malnutrition as determined by BCM.

## 1. Introduction

Many studies have reported the presence of malnutrition in a large fraction of hemodialysis (HD) patients (pts). The majority of these studies revealed that protein energy wasting was associated with increased morbidity, mortality, and impaired quality of life [1]. Several markers, such as low BMI (weight/height^2^), low serum albumin (Alb), low serum cholesterol (Chol), and elevated C-reactive protein (CRP) either as isolated metrics or incorporated as part of a score, have been previously associated with undernutrition in populations of HD pts [1].

The aims of this study were to analyse body composition (BC), to detect the presence of undernutrition, and to establish a relationship between undernutrition and the biological markers routinely used as indicators of nutritional status in HD patients from several HD units of Argentina.

We used a portable device (a body composition monitor (BCM Fresenius)) that expresses body weight in terms of lean tissue mass (LTM) and fat tissue mass (FTM) independent of hydration status.

The BCM model has recently been validated in multicentre studies against the respective gold standards both in healthy subjects and in HD pts [2–5].

The BCM Fresenius device uses an easy and noninvasive method that incorporates a novel three-compartment (3C) body composition model and involves bioimpedance spectroscopy (BIS), a technique that measures the impedance of body tissues over wide frequency ranges.

The BCM was first validated against gold standards to determine total body water (TBW), extracellular water (ECW), and intracellular water (ICW) [5] and then on the basis that in healthy subjects a given mass of tissue could be associated with fixed proportions of ICW and ECW regardless of BC.

The consequence of these fixed parameters is that the ratio ECW/ICW is constant in a specific tissue. Fixed hydration parameters allow excess fluid (ExF) to be identified by the new model using 3 whole body measurements of weight, ECW, and ICW. This 3C model differentiates normal hydrated (NH) LTM from NH FTM regardless of the degree of ExF and it offers a more reliable alternative to measure both hydration status and nutritional status which are usually altered in HD pts [6].

Based in this approach BCM defines ExF as overhydration (OH) with a normal range ±1, 1 liters (L) [7]. Recently published studies using the BCM have shown both a high prevalence of low LTM and overhydration to be associated with increased mortality among HD pts [8].

## 2. Material and Methods

From nine HD units of Buenos Aires and its suburbs, 934 pts were included (44% women and 17% diabetics). A total of 4200 patients were receiving chronic dialysis treatment in the Buenos Aires area at the time of the BCM study [9, 10]. The majority of patients were dialysed three times a week for ≥4 hours using low and high flux polysulfone membranes. The patients characteristic, parameters delivered by BCM, and measured variables are shown in Table 1.

Undernutrition was defined as having a lean tissue index (LTI = LTM/height^2^) below the 10th percentile of a reference population derived from BCM measurements of 1000 healthy adult subjects aged 18–75. This reference population is age and gender specific, as BC varies throughout life and between genders [11].

Exclusion criteria were dictated by the device and included history of a pacemaker, defibrillator, metallic sutures, or stent implantation and amputation of a major limb.

BC measurements were made with a portable whole body BIS device (BCM Fresenius Medical Care D GmbH).

Measurements were taken before the start of the HD treatment with the patient calm, supine, and relaxed in the dialysis chair for 2 minutes after the electrodes had been attached to the hand and foot on the same side of the body.

All measurements were performed by a renal dietician and/or a trained nurse.

Pts were separated into 2 groups: low LTI (LTI < 10th percentile of a reference population) [9] and normal LTI (LTI ≥ 10th percentile). Nutritional status markers, such as Alb (green bromocresol method, cut-off value = 4 g/dL), Chol (enzymatic colorimetric method, cut-off value = 200 mg/dL), CRP (immunoturbidimetry method, cut-off value = 6 mg/L), BMI, fat tissue index (FTI = FTM/height^2^), and time on dialysis (TD), were used to compare these groups. To assess fluid status we used a recently defined index (RelOH%) [7] in which the absolute volume overload (OH(L)) was normalized to the BCM-measured extracellular water (OH(L)/ECW(L)), a parameter denoted as “relative overhydration” (relative OH), expressed in percentage, to allow a more objective comparison between patients with different anthropometric features. RelOH% > 15% (severe OH) is an important and independent predictor of mortality in chronic HD patients [8].

The monthly laboratory data previous to the treatment involving the BCM measurement were recorded. All serum samples were processed in a central laboratory.

### 2.1. Statistical Analysis

A *P* value <0.05 was considered to be statistically significant in this study. Comparisons between different groups were performed using the Kruskal-Wallis test [12].

Correlation coefficients were determined using the Pearson method.

Receiver operating characteristics (ROC) analysis was performed to estimate the cut-off of continuous variables related to low LTI and high FTI.

In order to evaluate the discrimination characteristics of each marker, a ROC analysis was performed to select the best cut-off value. For each marker we spanned cut-off levels from minimum to maximum value (ten steps). In each step sensitivity (Se) and specificity (Sp) were calculated according to the BCM classification. The Se-Sp pair achieving the smaller distance to the optimal point (Sp = 100%, Se = 100%) was chosen as the optimal cut-off for this variable [13].

BMI, FTI, RelOH%, Alb, Chol, and CRP were included in the ROC analysis related to low LTI (undernutrition), while BMI, RelOH%, Alb, Chol, and CRP were included in the ROC analysis related to high FTI.

All values are expressed as mean ± standard deviation or median (range) as appropriate.

## 3. Results

58.8% of pts were malnourished, as defined by low LTI, while 57.8% of pts showed increased fat deposits, as defined by having FTI > 90th percentile of a reference population [11].

13.5% of the studied population and 23% of the low LTI group showed severe overhydration (RelOH% > 15%).

**Figure 1:**
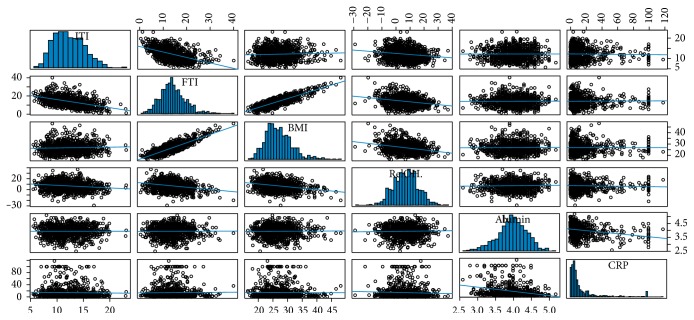
Bivariate scatterplots between body components (LTI (Kg/m^2^), FTI (Kg/m^2^), and BMI (Kg/m^2^)), hydration status (RelOH%), and nutritional biomarkers (albumin and C-reactive protein).

Table 2 and Figure 1 show a correlation between parameters delivered by BCM and biochemical markers. They demonstrate that LTI had an inverse correlation with FTI (*r* = −0.48) and RelOH% (*r* = −0.17). They also show a positive correlation between BMI and FTI (*r* = 0.84). However LTI did not show any relationship with BMI. The analysis of the relation LTI-FTI showed that in patients with highest BMI (>35) the growing of fat depots is independent of the behaviour of the LTI.

Low LTI pts were older and significantly overhydrated and had been on HD for a longer period of time (higher TD) than the normal LTI group (Table 3).

FTI was significantly higher in patients with low LTI (Table 3) and increased according to BMI (Table 4). LTI shows few changes related to increased BMI (Table 4 and Figure 1).

**Figure 2:**
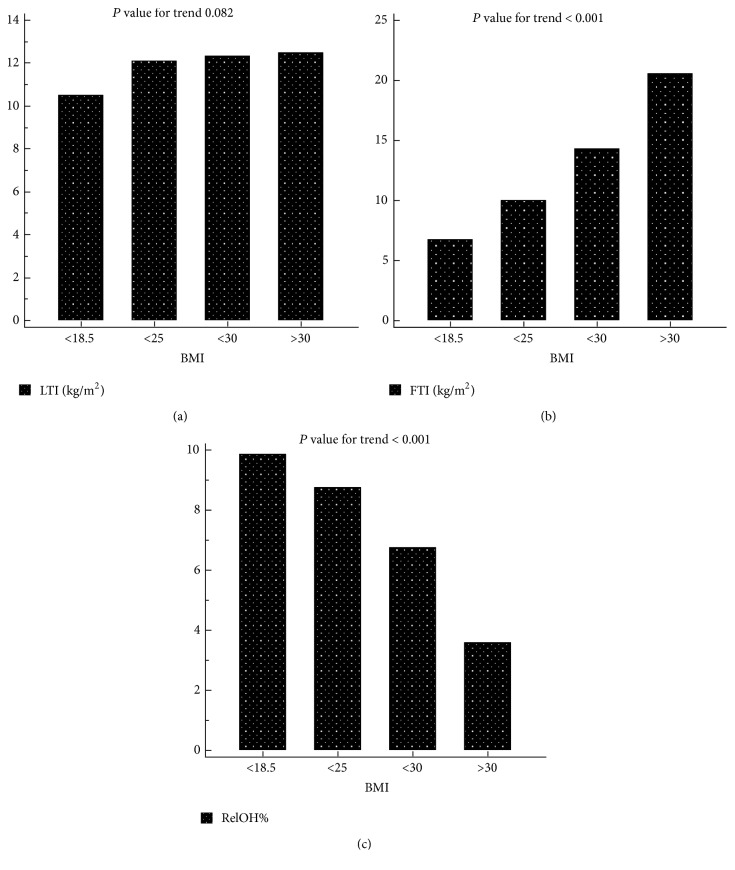
(a) Distribution of LTI expressed as mean according to BMI (WHO classification). (b) Distribution of FTI expressed as mean according to BMI (WHO classification). (c) Distribution of RelOH% expressed as mean according to BMI (WHO classification).

The prevalence of undernutrition, expressed as a low LTI, was more than fifty percent in all the BMI subgroups according to the World Health Organization's (WHO's) classification [14] (Table 4 and Figure 2). The subgroup of obese patients (*n* = 214) showed less overhydration (Table 4).

To analyse the association of low LTI and high FTI with the markers, ROC curves were calculated.

BCM-defined FTI and RelOH% showed an area under curve significantly different from line of reference with lower sensitivity and specificity to predict low LTI. But BMI, Alb, CRP, and Chol were unable to discriminate low LTI from normal LTI pts (Table 5). BMI with a cut-off of 25.8 yields a 79.2% sensitivity and 82.49% specificity strongly predicts increased fat deposits (Table 6).

Alb and CRP correlated inversely (*r* = −0.28). However neither of them was statistically different when considering undernourished and normal LTI pts (see Table 2 and Figure 1).

Table 7 shows the distribution of nutritional markers in the low LTI subpopulation (*n* = 550): 167 pts (30.36%) included in this group had normal (N) values of Alb (≥4 g/dL) and CRP (≤6 mg/L); 151 pts (27.45%) showed N values of Alb and increased values of CRP (>6 mg/L); 144 pts (26.18%) had lower values of Alb (<4 g/dL) and increased values of CRP (>6 mg/L) and 88 pts (16%) had lower values of Alb and normal values of CRP. The prevalence of high FTI was uniformly distributed in this subpopulation.

## 4. Discussion

Our BCM study was able to show a high prevalence of undernutrition, as expressed by low LTI, and was the first application of this BCM device in Argentina.

These findings are consistent with several other studies. Among these studies, the prevalence of undernutrition differed according to the method used to define nutritional status [1].

In the French national cooperative study [15], which included 7123 pts, nutritional status was determined by BMI, normalized protein catabolic rate, laboratory values, and LTM calculated from the creatinine generation rate. This study showed undernutrition in 20–36% of the studied population, according to nutritional parameters. A remarkable aspect of this study was the high prevalence (62%) of reduced LTM similar to our findings of around 60% of low LTI pts in a BCM approach. Other technologies, like DXA (dual X-ray absorptiometry) and BIA (bioelectrical impedance analysis), used for this purpose showed a high prevalence of low LTM [1]. Woodrow found a significantly lower LTM measured with DXA and BIA in pts with advanced chronic renal failure, peritoneal dialysis, and HD compared with a healthy group [16].

The low-LTI population showed significantly longer times in HD in this study. Chertow et al. [17] described that time on dialysis was associated with impaired nutritional status and increased mortality risk.

Older HD pts may be a greater risk for undernutrition than their younger counterparts [18]. In our study low LTI pts were significantly older than the normal LTI group. This was a relevant finding because the BCM device defines low LTI after comparing the measurement with a reference range derived from healthy controls according to gender and age [11].

The ability of BCM to differentiate lean tissue from fat mass allowed us to describe a strong association between FTI and BMI as well as low LTI and high FTI. Using DXA technology, Leinig et al. [19] reported strong and significant correlation between BMI and fat mass in a study of a small number of CKD stage 3–5, peritoneal, and HD pts. Torun et al. [20] arrived to similar conclusions in a BIA analysis.

In healthy adults, BMI is dependent on muscle and fat mass [14]. In our study, BMI clearly expressed the changes in the fat deposits but failed to show lean mass status. This situation was confirmed by the ROC curves and by the high prevalence of undernutrition in patients with BMI ≥ 23 kg/m^2^, a value commonly recommended as predictor of better survival in HD populations [1, 21].

Despite being an observational study of an unselected population, the conclusion was that increased fat deposits, especially those related to low LTM, are a characteristic pattern of body composition for this population. In a DXA-based study in which nutritional status was defined by subjective global assessment, Honda stated that protein energy wasting is not uncommon in overweight pre-HD pts and it was associated with high fat mass, low LTM, and inflammation, defining this situation as obese sarcopenia [22].

Albumin and CRP, both strongly related to inflammation-malnutrition [21], were shown to be poor predictors of malnutrition in this study. However, 40% of the study population showed hypoalbuminemia and 55% had elevated levels of CRP, showing an inverse relation between them (*r* = −0.28), which has been strongly associated with inflammation, cardiac disease, and poor outcomes in several studies [23, 24]. Also, analysis of the subpopulation with low LTI showed that only 30% of the pts had normal values of albumin and CRP (Table 7).

The high prevalence of overhydration in our population, and especially in low LTI pts, could explain some of the many factors to consider that could affect albumin levels [25].

Recently, a study using BCM technology to define hydration status showed that hyperhydration is an independent predictor of mortality [8].

Another interesting finding in our population was the inverse relation of LTI-FTI and overhydration (RelOH%); in our study, pts with higher BMI (defined as overweight and obese according WHO) showed less overhydration and higher fat mass (Table 4) findings that could match classical theory of reverse epidemiology [26]. In our study lean mass did not change significantly with higher BMI. Some studies [27] showed that a survival advantage of higher fat mass appeared to be superior to that of lean body mass.

Even though we did not include clinical outcomes in our study, the high prevalence of undernutrition and fluid overload could have a strong impact in outcome and clinical practice.

A recently published study of a Slovakian population showed that low LTI was a strong independent predictor of mortality where intervention (nutritional supplements) might improve nutrition and reduce the mortality risk [28].

Also these findings may explain the multifactorial aetiology of undernutrition in this HD population related to low protein intake [15], inflammation [23], overhydration [8], low physical activity [29], and ageing [18].

Metallic endovascular devices, pacemakers, or amputations are exclusion criteria for BCM study in an HD population in which cardiovascular burden is quite frequent though it could result in a potential bias. Therefore, early detection of muscle mass loss and overhydration in pts with CKD stages 3–5 with BIS approach could help to change history of the disease. Szu-Chun Hung showed a higher prevalence of volume overload, undernutrition, and inflammation in CKD stage 3–5 pts diabetics as well as diabetics [30].

Implementation of BCM technology as a bed-side clinical tool in HD pts was easy and noninvasive and should be considered for helping the health team in his decision making.

## 5. Conclusions

In our study, BMI and other common markers, such as albumin, failed to predict malnutrition as determined by BCM.

Trend analysis based on repetitive BCM measurements (evaluating intraindividual variability) should be considered as an earlier marker of undernutrition in HD pts in which traditional anthropometrics, somatic proteins, and inflammation biomarkers showed a high variability.

Future BCM-based studies in our country that include a follow-up of the cohort may be able to show the true significance of the high prevalence of increased fat deposits with and without low LTI and hydration status in this population.

## Figures and Tables

**Table 1:** The patients characteristic, parameters delivered by BCM, and measured variables: 25–75 P (25th–75th percentiles).

	Mean	SD	Median	25–75 P
BMI (kg/m^2^)	26.8	4.7	26.1	23.5–29.7
FTI (kg/m^2^)	14	5.5	13.5	10.4–17.2
LTI (kg/m^2^)	12.2	3.0	12	9.9–14.4
Height (cm)	162.5	9.9	162	155–169
Weight (kg)	71.2	15.5	69.1	60–80
Age (yrs)	58	15.8	60	46.8–70.1
TD (yrs)	5.5	5.1	3.8	21–97.2
Albumin (gr/dL)	4.02	0.41	4	3.8–4.3
Cholesterol (mg/dL)	171.4	40.8	165	142–198
CRP (mg/L)	14.1	20.6	6.6	3.7–15.1
RelOH%	6.8	9.1	6.9	7.1–12.8

**Table 2:** Correlation between BCM delivered parameters and nutritional biomarkers.

	BMI (kg/m²)	FTI (kg/m²)	LTI (kg/m²)	RelOH (%)	Albumin	Cholesterol	CRP
FTI (kg/m²)	**0.84** **<0.0001**		**−0.48** **<0.0001**	**−0.24** **<0.0001**	0.01 0.8561	−0.06 0.0812	0.01 0.6642
LTI (kg/m²)	0.06 0.0908	**−0.48** **<0.0001**		**−0.17** **<0.0001**	0.01 0.8538	0.06 0.0757	−0.02 0.4845

**Table 3:** Variables related to nutritional status (mean (SD)).

*n*	Low LTI (550)	Normal LTI (384)	*P*
BMI (kg/m^2^)	26.6 (4.7)	27.1 (4.7)	0.0204
Albumin (gr/dL)	4.01 (0.41)	4.02 (0.41)	0.3892
Cholesterol (mg/dL)	170 (39.4)	173.4 (42.6)	0.3411
CRP (mg/L)	14.7 (22.3)	13.6 (17.9)	0.3093
FTI (kg/m^2^)	15.3 (5.3)	12.1 (5.1)	<0.0001
TD (yrs)	6.1 (6)	4.9 (4.7)	<0.0001
Age (yrs)	58.9 (15.8)	55.5 (15.7)	0.004
RelOH%	8.2 (9.2)	4.8 (8.3)	<0.0001

**Table 4:** Distribution of LTI and FTI and overhydration (RelOH%) expressed as mean (SD) according to BMI (WHO classification).

BMI (*n*)	<18.5 (11)	18.5–24.9 (354)	25–29.9 (355)	≥30 (214)	*P* value for trend
FTI (kg/m²)	6.75 (1.94)	9.97 (3.34)	14.32 (3.31)	20.52 (4.79)	<0.001
LTI (kg/m²)	10.5 (1.95)	12.08 (2.96)	12.32 (3.03)	12.48 (3.15)	0.082
RelOH%	9.81 (11.11)	8.73 (8.87)	6.74 (8.77)	3.57 (8.87)	<0.001

**Table 5:** Area under the curve and cut-off values from ROC curves of BMI, albumin, cholesterol, CRP, FTI, and RelOH% as predictors of low LTI.

	Cut-off	AUC	Sensitivity (%)	Specificity (%)
BMI (kg/m^2^)	26.1	0.543	54	55.73
Albumin (g/dL)	3.7	0.516	24.39	81.50
Cholesterol (mg/dL)	183	0.517	66.23	40.27
CRP (mg/L)	7.55	0.519	57.44	48.36
FTI (kg/m^2^)^**^	13.9	0.677	56.18	68.75
RelOH%^**^	7.94	0.611	53.27	63.28

^**^Significantly different from AUC 0.5 *P* < 0.001.

**Table 6:** Area under the curve and cut-off values from ROC curves of BMI, albumin, cholesterol, CRP, and RelOH% as predictors of high FTI.

	Cut-off	AUC	Sensitivity (%)	Specificity (%)
BMI (kg/m^2^)^**^	25.8	0.894	79.81	82.49
Albumin (g/dL)	4.39	0.52	22.77	82.32
Cholesterol (mg/dL)	134	0.507	18.7	84.49
CRP (mg/L)	3.98	0.504	29.75	74.47
RelOH%^**^	6.36	0.595	55.93	61.17

^**^Significantly different from AUC 0.5 *P* < 0.001.

**Table 7:** Distribution of nutritional markers in patients with low LTI.

	Alb N C-RP N	Alb N C-RP ↑	Alb ↓ C-RP ↑	Alb ↓ C-RP N
*n*	167	151	144	88
High FTI (%)	69	69	66	63

Alb: N ≥ 4 g%; ↓  <  4 g%. C-RP: N ≤ 6 mg/L; ↑  >  6 mg/L.
